# Identifying Predictive Biomarkers of Response in Patients With Rheumatoid Arthritis Treated With Adalimumab Using Machine Learning Analysis of Whole‐Blood Transcriptomics Data

**DOI:** 10.1002/art.43255

**Published:** 2025-08-04

**Authors:** Chuan Fu Yap, Nisha Nair, Ann W. Morgan, John D. Isaacs, Anthony G. Wilson, Kimme Hyrich, Guillermo Barturen, María Riva‐Torrubia, Marta Gut, Ivo Gut, Marta E. Alarcón Riquelme, Anne Barton, Darren Plant

**Affiliations:** ^1^ Centre for Genetics and Genomics Versus Arthritis, Centre for Musculoskeletal Research, The University of Manchester Manchester United Kingdom; ^2^ Centre for Genetics and Genomics Versus Arthritis, Centre for Musculoskeletal Research, The University of Manchester and NIHR Biomedical Research Centre Manchester Manchester United Kingdom; ^3^ School of Medicine University of Leeds and NIHR Leeds Biomedical Research Centre, Leeds Teaching Hospitals NHS Trust Leeds United Kingdom; ^4^ Translational and Clinical Research Institute, Newcastle University and NIHR Newcastle Biomedical Research Centre and Musculoskeletal Unit, Newcastle‐upon‐Tyne Hospitals NHS Foundation Trust, Newcastle‐upon‐Tyne United Kingdom; ^5^ School of Medicine and Medical Science, Conway Institute, University College Dublin Dublin Ireland; ^6^ NIHR Biomedical Research Centre Manchester and Centre for Epidemiology Versus Arthritis, Centre for Musculoskeletal Research, The University of Manchester Manchester United Kingdom; ^7^ GENYO, Centre for Genomics and Oncological Research Pfizer, University of Granada, Andalusian Regional Government, Parque Tecnológico de la Salud (PTS) Granada and Department of Genetics, Faculty of Science University of Granada and Bioinformatics Laboratory, Biotechnology Institute, Centro de Investigación Biomédica, PTS Granada Spain; ^8^ Centre for Genomics and Oncology Research (GENYO), Centre for Genomics and Oncological Research Pfizer, University of Granada, Andalusian Regional Government, PTS Granada Granada Spain; ^9^ CNAG and Universitat de Barcelona Barcelona Spain; ^10^ GENYO, Centre for Genomics and Oncological Research Pfizer, University of Granada, Andalusian Regional Government, PTS Granada, Granada, Spain, and Institute for Environmental Medicine, Karolinska Institute Stockholm Sweden

## Abstract

**Objective:**

Tumornecrosis factor inhibitors (TNFi) have significantly improved rheumatoid arthritis (RA) management, yet variability in patient response remains a substantial challenge, with approximately 40% of patients discontinuing TNFi due to nonresponse or adverse effects. This study aimed to identify biomarkers predictive of adalimumab treatment response using whole‐blood transcriptomics, leveraging machine learning models for data mining observed by targeted statistical analysis.

**Methods:**

A cohort of patients with RA starting TNFi therapy (n = 100) was assessed for treatment response at 6 months, with RNA sequencing performed on baseline (pretreatment) and 3‐month follow‐up samples. Machine learning classifiers were built to identify predictive biomarkers for treatment outcomes. This was observed by a network analysis on the biomarkers to elucidate the most influential biomarker, which was subsequently confirmed through survival analysis.

**Results:**

Differential gene expression analysis in 97 samples passing quality control identified 84 genes associated with treatment response. Random forest classifiers achieved high predictive accuracy with area under the receiver operating characteristic curves up to 0.86, identifying genes contributing to treatment outcomes. Network analysis further elucidated gene interactions, highlighting marginal zone B And B1 cell–specific protein 1 (*MZB1*) as a novel biomarker not captured by machine learning alone. MZB1's role in B cell development and antibody production was associated with antidrug antibody formation, impacting treatment efficacy.

**Conclusion:**

This study advances the understanding of transcriptomic alterations in RA treatment and enhances our understanding of treatment response mechanisms. Although the gene signatures identified require independent replication, the study serves as a starting point to pave the way for personalized therapeutic strategies in patients commencing TNFi therapy in RA.

## INTRODUCTION

Tumor necrosis factor inhibitors (TNFi) have significantly improved the management of rheumatoid arthritis (RA), offering relief from joint inflammation and the associated risk of cartilage and bone damage. However, variability in patient response remains a significant challenge, with approximately 40% of individuals discontinuing TNFi due to primary nonresponse, loss of response, or unwanted side effects of these therapies.^1^

Several clinical and demographic factors (for example, age, sex, disease duration, and concurrent conventional synthetic disease‐modifying antirheumatic drugs [csDMARDs]) provide some insight into treatment response variability. However, these factors are not sufficiently predictive to be useful clinically,^2^, ^3^, ^4^, ^5^, ^6^ and they do not inform on relevant underlying biologic pathways that underpin variation in response. Identifying biologic predictors or biomarkers of treatment response, ideally before starting TNFi therapy, is therefore a research priority, would enable informed therapeutic decision‐making, and would provide better understanding of the mechanisms of response.^7^

Previous TNFi response studies in RA incorporating transcriptomics data have yielded encouraging results. For example, Oswald et al identified consistent changes in predefined gene expression modules that were associated with a good response in whole‐blood transcriptomics data across three independent US‐based RA cohorts.^8^, ^9^ These results were later replicated in a UK cohort of patients with RA.^9^ Blood‐based transcriptomics profiles have also been used to classify treatment response with good‐to‐moderate performance as a single data modality^10^, ^11^ or as part of a multiomics approach.^12^, ^13^, ^14^, ^15^ However, many previous studies were based on transcript arrays with limited coverage and dynamic range of expression values, were performed using a single sampling timepoint, analyzed pooled patients on different medications, or used statistical models that were not developed under robust nested cross‐validation to mitigate overfitting. More recently, transcriptomics profiling of synovial tissue has revealed interesting biology in studies of rituximab and tocilizumab; however, tissue is substantially more challenging to acquire and process compared to blood samples, and the predictive ability of transcriptomics data in this setting was limited, meaning additional model refinement is needed.^16^

In the current study, we employ refined analytical techniques to investigate the transcriptomic alterations in the whole blood of patients with RA treated with adalimumab. We used next‐generation sequencing technologies to provide a more detailed view of gene expression patterns along with state‐of‐the‐art machine learning and network analysis. This approach allows for a comprehensive examination of the transcriptome to identify novel biomarkers that associate with the response of patients with RA to TNFi therapy.

## METHODS

### Patient cohort

The patient samples used in this study were available from patients recruited to the Biologics in Rheumatoid Arthritis Genetics and Genomics Study Syndicate (BRAGGSS, Research Ethics Committee reference 04/Q1403/37), a prospective multicenter observation study cohort based in the United Kingdom. BRAGGSS patients included in the current study had a diagnosis of RA according to the American College of Rheumatology 1987 revised criteria for the classification of RA,^17^ were of European ancestry, and were about to receive treatment with adalimumab for their RA symptoms. Adalimumab was chosen because it was the most commonly prescribed TNFi for RA in the United Kingdom at the time of study design. Patients were selected for this study based on (1) being about to commence treatment with adalimumab and (2) the availability of paired blood samples for bulk transcriptomics at baseline and a 3‐month follow‐up. The samples for the current study were then selected at random from the BRAGGSS samples with this information available. Treatment response was assessed at 6 months after starting therapy using the EULAR criteria,^18^ categorizing patients into good, moderate, and nonresponders based on changes to the scores of the Disease Activity Score in 28 joints using the C‐reactive protein level (DAS28‐CRP). There were four patients whose DAS28‐CRP scores were not available for the 6‐month timepoint; for these patients, the 3‐month DAS28‐CRP score was used for computing the EULAR response. For analytical purposes, good and moderate responders were grouped as “responders.”

### RNA sequencing

Total RNA samples were quality controlled using the Qubit RNA Broad‐Range Assay Kit (Thermo Fisher Scientific) to assess quantity and the Fragment Analyzer DNF‐471 RNA Kit (15 nt) (Agilent Technologies) to evaluate integrity. RNA‐sequencing (RNA‐seq) libraries were generated from total RNA using the TruSeq Stranded messenger RNA (mRNA) Library Preparation Kit (Illumina). In brief, mRNA was enriched from 500 ng of total RNA using oligo‐dT magnetic beads. The resulting blunt‐ended double‐stranded complementary DNA was 3' adenylated, and Illumina platform‐compatible adapters with unique dual indexes and unique molecular identifiers (Integrated DNA Technologies) were ligated. The ligation product was enriched with 15 polymerase chain reaction cycles. The final library was validated using a Bioanalyzer DNA 7500 assay (Agilent Technologies). Libraries were sequenced on a NovaSeq 6000 (Illumina) in paired‐end mode with a read length of 2 × 151 bp, following the manufacturer's protocol for dual indexing. Image analysis, base calling, and quality scoring were processed using the manufacturer's software Real‐Time Analysis (version 3.4.4), observed by the generation of FASTQ sequence files. The alignment of sequencing reads to the human reference genome (GENCODE version 39 hg38) was performed using Spliced Transcripts Alignment to a Reference software.^19^ Quantification of gene expression levels was undertaken using RNA‐seq by Expectation‐Maximization.^20^

### Differential expression analysis

Initial filtering removed genes with cumulative counts below 500 across samples and those expressed in fewer than 20% of samples. Differential gene expression analysis was conducted using the generalized linear mixed model with negative binomial distribution through glmmSeq R package,^21^ controlling for patient variability as a random effect. Dispersion parameters, θ, were estimated using DESeq2.^22^ Interaction terms for timepoint and treatment response were incorporated alongside sex and baseline DAS28‐CRP scores as covariates, which results in the following model:

$$\;\mathrm{yij}\sim \mathrm{NB}\left(\mu_{\mathrm{ij}},\theta \right)$$

$$\log\left(\mu_{\mathrm{ij}}\right)=\begin{array}{l} \beta_{0}+\beta_{1}{\mathrm{Sex}}_{i}+\beta_{2}\text{BaselineDAS}28{\mathrm{CRP}}_{i}+\beta_{3}{\text{Response}}_{\mathrm{ij}}\\ +\;\beta_{4}{\text{Timepoint}}_{\mathrm{ij}}+\beta_{5}\left({\text{Timepoint}}_{\mathrm{ij}}\times {\text{Response}}_{\mathrm{ij}}\right)+u_{i} \end{array}$$

Where y_ij_ is the gene expression for patient I at observation j; β_0_ is the intercept; β_1_ and β_2_ are fixed‐effect coefficients for the covariates sex and baseline DAS28‐CRP scores, respectively; β_3_, β_4_, and β_5_ are fixed‐effect coefficients for response, timepoint, and their interaction, respectively; and u_i_ ∼ N(0, $\sigma_{u}^{2}$) is the random‐effect term for patient variability. Genes meeting a false discovery rate (FDR) below 0.05 and a fold change greater than 1.2 in either interaction or treatment response coefficient were classified as differentially expressed.

### Machine learning

The variance‐stabilizing transformation (VST) from DESeq2 was applied to the gene expression dataset, normalizing variance across genes. The confounding variables of sex and baseline DAS28 scores were accounted for by regression modeling, using linear models through statsmodels library^23^ on the VST data, with the resultant residuals used as input for the machine learning classifier. The random forest (RF) classifier models were trained using the scikit‐learn (sklearn) Python library.^24^ For RF training, a pipeline was employed in which feature selection was first performed with glmmSeq to identify differentially expressed genes (DEG). These DEGs were then used as input for the RF classifier as the next step in the pipeline. DEGs were determined in the outer loop of a nested 10‐fold cross‐validation method, with hyperparameter optimization performed within the five‐fold nested inner loops. To optimize the hyperparameters of the RF model, a search was conducted using RandomizedSearchCV. The parameter search space included the number of estimators (n_estimators) ranging from 1,000 to 5,000 in increments of 200; the maximum tree depth (max_depth) ranging from 100 to 1,000 in increments of 100; the minimum samples required to split an internal node (min_samples_split) ranging from 0.1 to 1.0 in increments of 0.1; the minimum samples required to be at a leaf node (min_samples_leaf) ranging from 0.01 to 0.5 in increments of 0.05; and the number of features considered for splitting (max_features), which included options “sqrt” and “log2” and fractions of the total features (0.1, 0.2, 0.3, 0.4, and 0.5). The bootstrap sampling parameter (bootstrap) was also included in the search, with the options “True” and “False”. This approach facilitated a comprehensive exploration of the parameter space to identify the optimal configuration for maximizing model performance while maintaining computational efficiency. Three models were explored: inputs from (1) baseline‐only data, (2) follow‐up data only, and (3) data from both timepoints. Model performance was measured using the area under the receiver operating characteristic curve (AUC). Feature importance was assessed by the mean decrease in impurity within the RF, averaged over 1,000 iterations to account for variability in this stochastic process. The final models with the best hyperparameters identified were trained on the entire dataset, and only the top 30 significant genes, defined as features that contributed to at least 1% of feature importance, were selected for functional enrichment analysis using Metascape.^25^

### Network analysis

Networks of the top 30 most influential genes were constructed employing the graphical lasso (GLASSO) technique,^26^ with network parameters trained via sklearn's GraphicalLasso estimator. Regularization strength (alpha) was fine‐tuned through 10‐fold cross‐validation, using naive multivariate normal Bayes classifier to maximize AUC scores for both training and validation sets. The network was visualized and interrogated using network.^27^ GLASSO was chosen for its use in discerning key connections amid high‐dimensional data noise, as opposed to traditional correlation networks.^28^

### Antidrug antibody assessment

Presence of antidrug antibodies (ADAs) against adalimumab were determined by radioimmunoassay (performed by Sanquin Diagnostic Services). Serum samples collected at 3, 6, and 12 months (where available) were analyzed, and a positive antiadalimumab titer set was defined as >12 arbitrary units/mL as previously described.^29^ Once a patient developed ADA at any time in the study, they were classed as ADA positive.

### Cox regression model

The Cox proportional hazards model was used to determine the time to immunogenicity event associated with gene expression level after transformation using the VST method from DESeq2. The model was implemented through lifelines Python package.^30^ The model was adjusted for biologic sex, age, and csDMARD use.

### Cell deconvolution analysis

Cell deconvolution was conducted using CIBERSORTx.^31^ RNA‐seq data were normalized using transcripts per million, and LM22 reference panel was used. Analysis was performed in relative mode with 1,000 permutations, and batch correction was performed using B‐mode. The output from CIBERSORTx was transformed using centered log ratio (CLR) transformation. A generalized linear model was used to model response outcome with CLR‐transformed cell type as a predictor. This was observed by multiple testing correction with Benjamini‐Hochberg FDR.

### Data availability

Data are available upon request. Sharing will be done in line with University of Manchester–recommended procedures and funding requirements. Our ethics (North West 6 Central Manchester South Research Ethics Committee [COREC 04/Q1403/37]) allows sharing of data with other bona‐fide researchers both in the United Kingdom and abroad. Model objects trained in this study will be made available on request.

## RESULTS

### Patient cohort

One hundred patients from within the BRAGGSS cohort were included in the current study. Following quality control of the RNA‐seq data, 97 samples remained available for analysis. During quality control for RNA sequencing, it was found that three samples (two responders and one nonresponder) were of low quality and were therefore not sequenced. A total of 72% of the participants were classified as responders following 6 months of therapy, and a significant portion were women (86.6%), reflecting the demographics of the wider population with RA (Table 1). A large proportion of the cohort used concurrent csDMARDs (85.6%) and with the majority using methotrexate co‐therapy (75.3%). Steroid usage was 14.6%, with similar response rates between users and nonusers. The baseline DAS28 was approximately 5.6, and the median age of participants was approximately 60 years, with minor differences between responders and nonresponders. Univariate statistical tests found no significant difference in these parameters between those who responded to the treatment and those who did not. Anti‐citrullinated protein antibody (ACPA) status was measured using the cyclic citrullinated peptide (CCP)‐2 assay for 31 of the patients within this study, and 90% of them were positive.

**Table 1:** Characteristics of patients with rheumatoid arthritis participating in the study*

Characteristics	Overall (n = 97)	Nonresponder (n = 27)	Responder (n = 70)
Female sex, n (%)	84 (86.6)	24 (88.9)	60 (85.7)
Concurrent csDMARD usage, n (%)	83 (85.6)	22 (81.5)	61 (87.1)
Age, median (Q1–Q3)	60.6 (51.8–65.1)	59.2 (48.9–64.6)	61.3 (55.1–65.0)
MTX co‐therapy, n (%)	73 (75.3)	22 (81.5)	51 (72.9)
Steroid usage, n (%)	14 (14.6)	4 (14.8)	10 (14.5)
Comorbidity present, n (%)	22 (22.7)	6 (22.2)	16 (22.9)
Baseline DAS28, mean (SD)	5.6 (0.7)	5.5 (0.8)	5.6 (0.7)
Disease duration, median (Q1–Q3)	4.7 (2.2–12.1)	5.9 (2.5–10.2)	4.6 (2.1–15.3)
Positive CCP status, n (%)	29 (90.6)	3 (75.0)	26 (92.9)
Adalimumab monotherapy, n (%)	12 (12.4)	5 (18.5)	7 (10.0)
Adalimumab + MTX, n (%)	52 (53.6)	15 (55.6)	37 (52.9)
Adalimumab + MTX + steroids, n (%)	10 (10.3)	3 (11.1)	7 (10.0)
Adalimumab + multiple csDMARDs, n (%)	19 (19.6)	3 (11.1)	16 (22.9)

* CCP, cyclic citrullinated peptide; csDMARD, conventional synthetic disease‐modifying antirheumatic drugs (sulfasalazine or hydroxychloroquine); DAS28, Disease Activity Score in 28 joints; MTX, methotrexate; Q1, first quartile; Q3, third quartile.

### Differential expression analysis of treatment response

**Figure 1:**
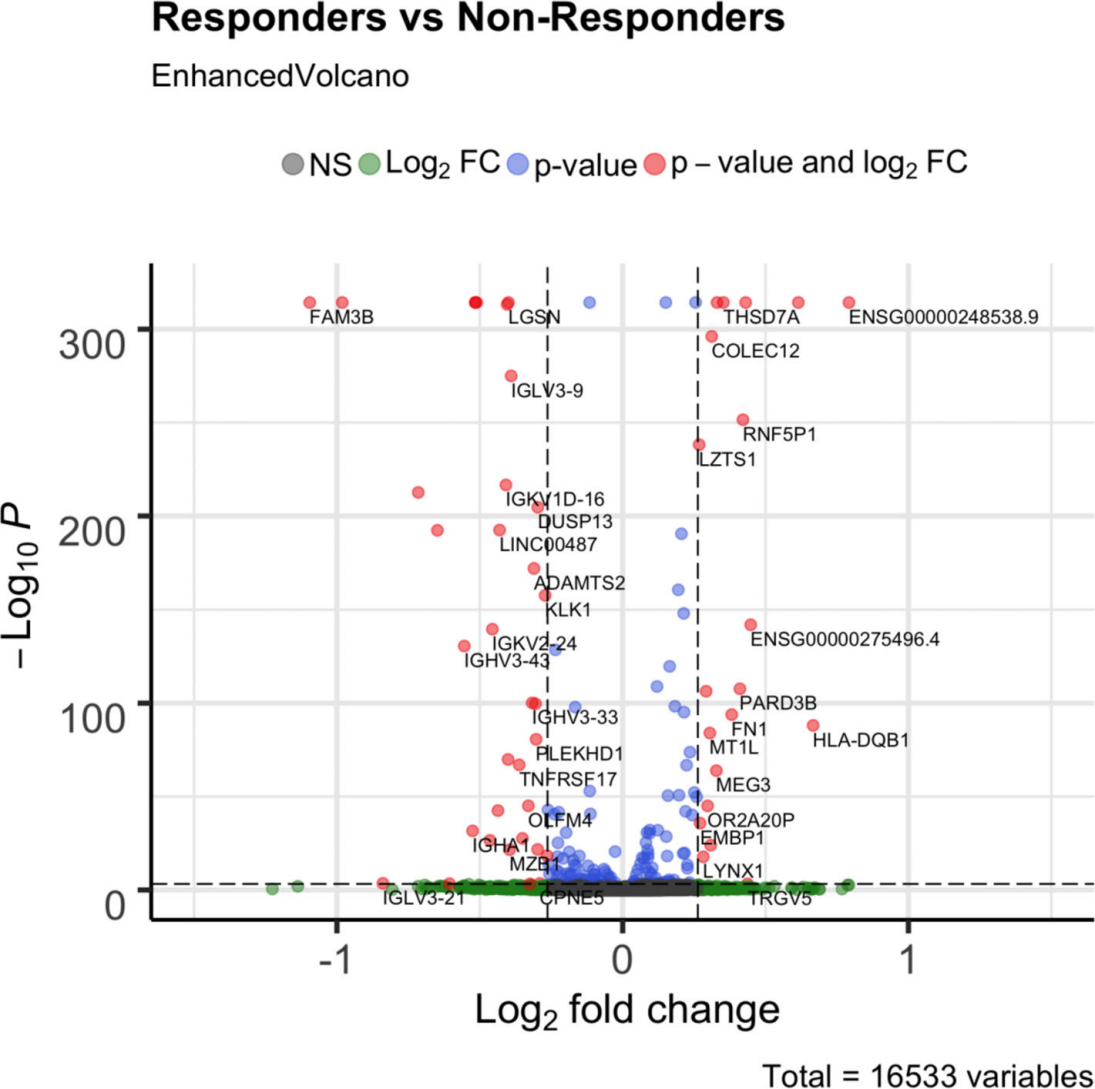
Volcano plot of glmmSeq model highlighting some of the differentially expressed genes. Log_2_ fold change of more than zero represents genes up‐regulated by responders or down‐regulated by nonresponders. Log_2_ fold change of less than zero represents genes down‐regulated by responders or up‐regulated by nonresponders. Horizontal dashed lines represent the cutoff for differentially expressed genes in terms of *P* value, whereas the vertical dashed lines represent log fold change cutoff. FC, fold change.

After filtering, 16,533 genes remained available for analysis. Eighty‐four genes showed differential expression between responders and nonresponders to adalimumab treatment (Figure 1). Thirty‐six genes had higher expression in responders compared with nonresponders, 50 genes were up‐regulated posttreatment (Figure S1), and 52 genes had a significant interaction effect (Figure S2); that is, expression of those genes between pretreatment and posttreatment differ between the two responder groups, either in the direction of effect or in the magnitude of change posttreatment. These results were all obtained from the full model with glmmSeq's negative, binomial, mixed‐effects model, which models response outcome and timepoint of sampling, as well as the interactions. Notably, *FAM3* metabolism regulating signaling molecule B (*FAM3B*) was the most down‐regulated gene in nonresponders, with a fold change of 0.46, whereas leiomodin 1 (*LMOD1*) was the most up‐regulated, showing a fold change of 1.53 (Figure 1). *HLA‐DQA1* and *HLA‐DQB1* associations with treatment response were also captured in this analysis with fold changes of 1.23 and 1.59, respectively (Figure 1). Gene set enrichment analysis was also carried using preranked method (Figure S3–S8), with pathways‐related UV radiation and protein secretion enriched for responders and pathways related to cell cycles and oxidative phosphorylation enriched posttreatment.

### Machine learning models identify predictive genes for treatment efficiency

Machine learning models were developed to predict treatment outcomes to identify genes with predictive potential. Three distinct models—baseline, follow‐up, and combined timepoints—were evaluated using nested 10‐fold cross‐validation. Given the high dimensionality of the transcriptomics data (>16,000 transcripts), the feature space was reduced during model development using differential expression analysis in the outer loop of the nested cross‐validation method.

The RF algorithm was selected for its robustness in handling tabular data, capacity to capture complex gene interactions, and interpretability of the algorithm. The model incorporating combined timepoints yielded an AUC of 0.77 (±0.18) for test sets. However, stratified models focusing on individual timepoints exhibited enhanced performance, with AUCs of 0.8 (±0.2) and 0.81 (±00.16) on the test sets for the pretreatment and 3‐month follow‐up data, respectively.

**Figure 2:**
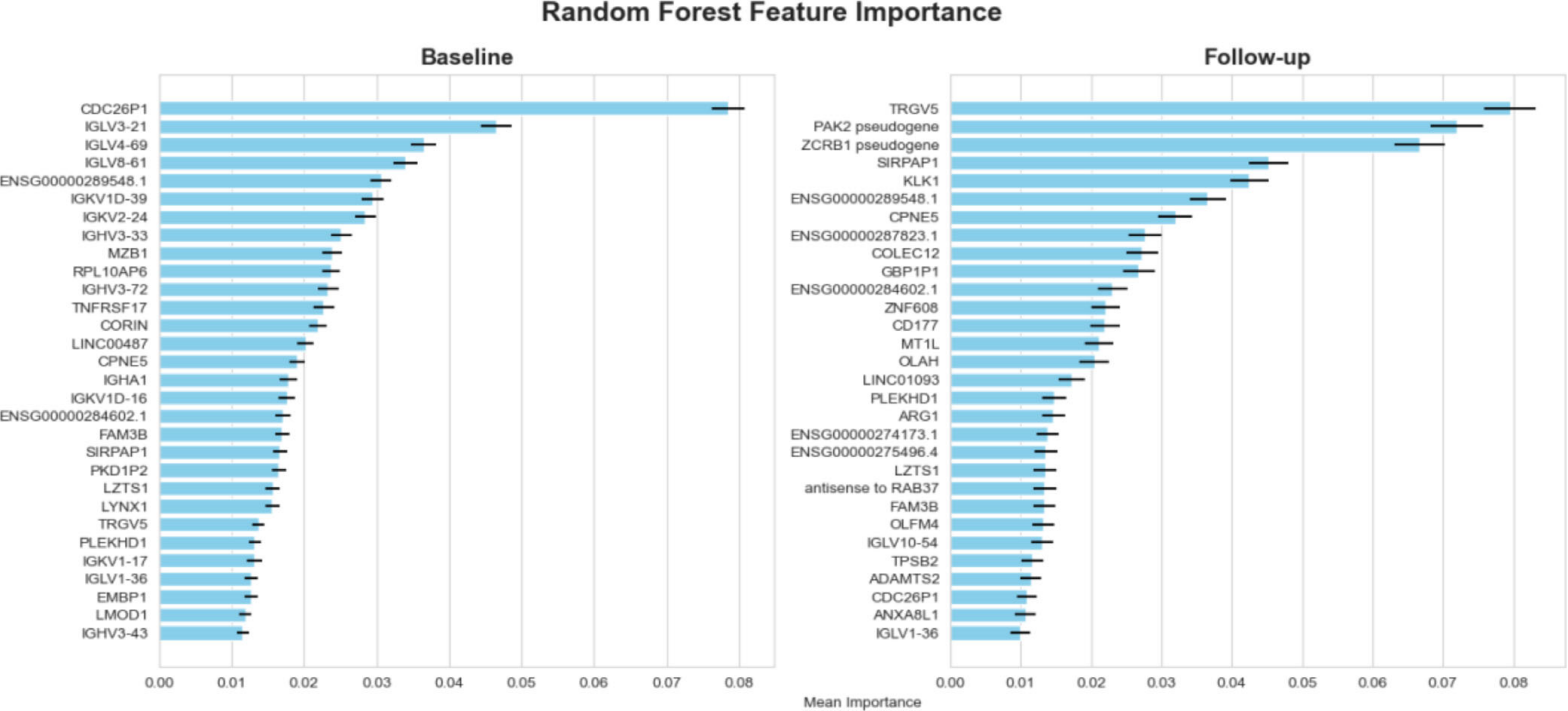
Feature importance for random forest classifiers generated based on mean decrease in impurity. Left is baseline classifier, and right is follow‐up classifier. The horizontal error bar is the SD resulting from 1,000 runs of random forest assessment. Color figure can be viewed in the online issue, which is available at http://onlinelibrary.wiley.com/doi/10.1002/art.43255/abstract.

**Figure 3:**
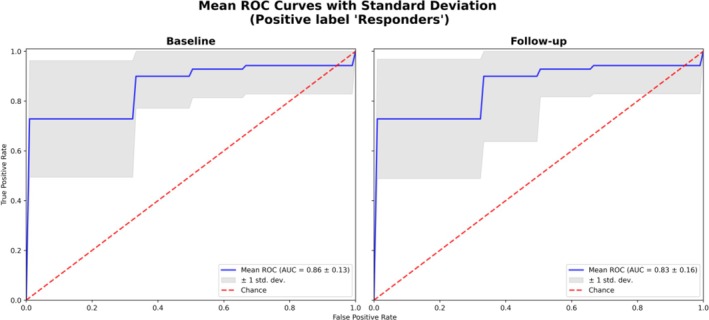
Receiver operating characteristic curve for baseline classifier (left) and follow‐up classifier (right). ROC, receiver operating characteristic; AUC, Area Under the Curve; std. dev., Standard Deviation. Color figure can be viewed in the online issue, which is available at http://onlinelibrary.wiley.com/doi/10.1002/art.43255/abstract.

Following hyperparameter optimization, the top 30 DEGs (Figure 2) identified using the full dataset were used to fit the final model, in which the baseline model achieved an AUC of 0.86 ± 0.13, and the final follow‐up model achieved an AUC of 0.83 ± 0.16 (Figure 3). There was modest overlap in the top 30 genes between baseline and follow‐up classifier with only 10 genes in common. The most important gene for the baseline classifier was cell division cycle 26 pseudogene 1 (*CDC26P1*), and for the follow‐up classifier, it was T cell receptor gamma variable 5 (*TRGV5*). For the baseline model, 16 of the top genes were Ig, and 50% of the follow‐up model top genes had no annotation (represented by Ensembl IDs in Figure 2). Many of these genes were noncoding RNA transcripts or pseudogenes.

Functional enrichment analyses at baseline indicated that the top 30 genes from the RF model were predominantly associated with the adaptive immune response (*P* = 1.94 × 10^−14^; Figure S9 and S10). A general down‐regulation was observed in good responders, with Ig genes constituting 12 of these genes. Conversely, the 3‐month timepoint showed enrichment in the innate immune response (*P* = 1.47 × 10^−3^), displaying a mix of up‐ and down‐regulation among good and poor responders.

### Network analysis elucidates gene interactions

**Figure 4:**
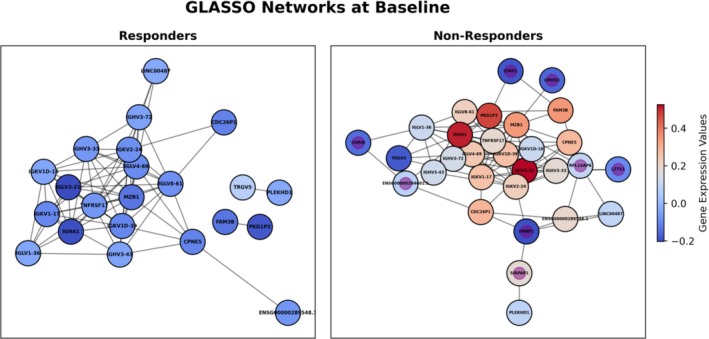
Connected component of GLASSO network for baseline, with responders on the left and nonresponders on the right. Nodes are colored by mean relative gene expression values, where blue is lower mean expression and red is higher mean expression. Nodes found only in nonresponders are marked with a purple center in the node. GLASSO, graphical LASSO. Color figure can be viewed in the online issue, which is available at http://onlinelibrary.wiley.com/doi/10.1002/art.43255/abstract.

**Figure 5:**
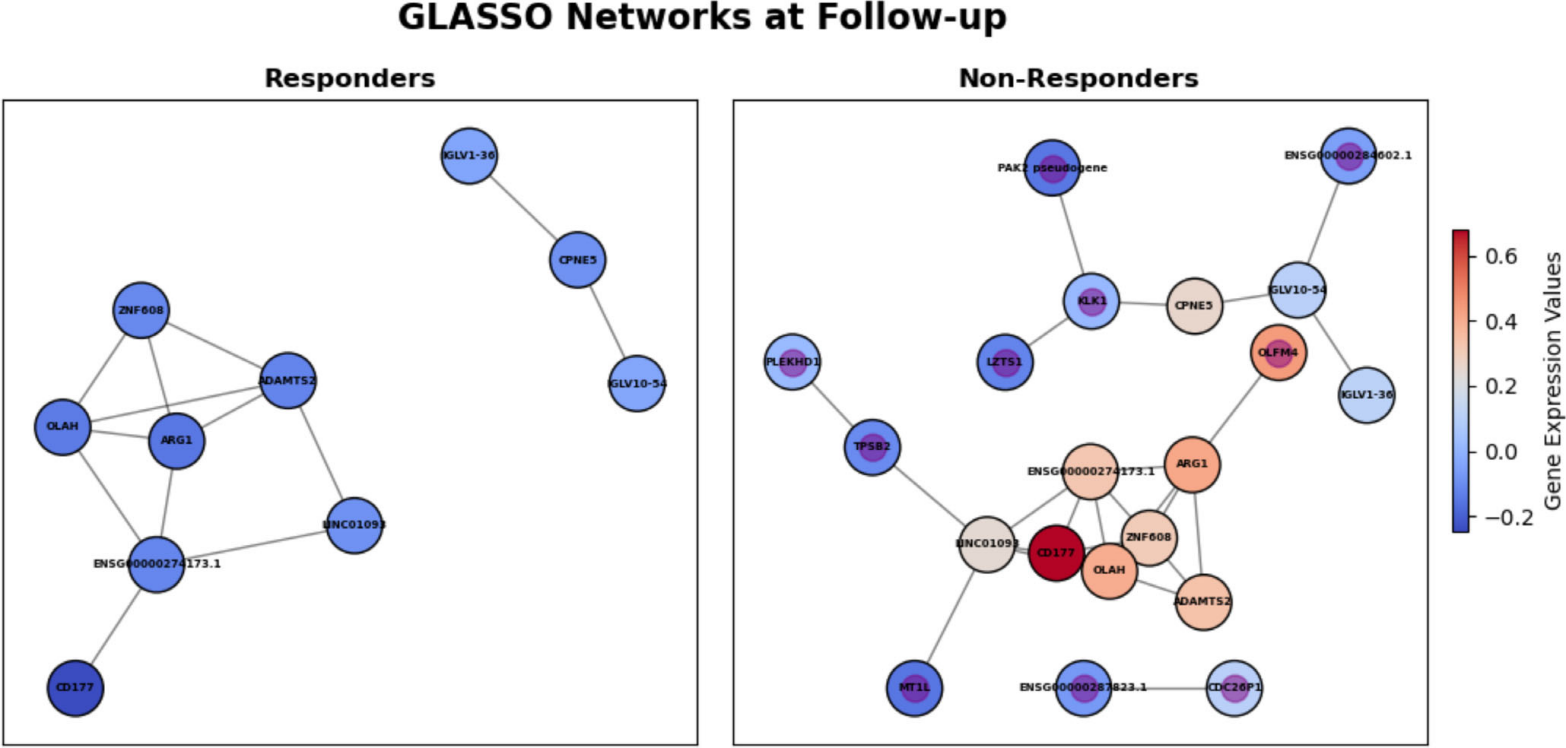
Connected component of GLASSO network for follow‐up, with responders on the left and nonresponders on the right. Nodes are colored by mean relative gene expression values, where blue is lower mean expression and red is higher mean expression. Nodes found only in nonresponders are marked with a purple center in the node. GLASSO, graphical lasso. Color figure can be viewed in the online issue, which is available at http://onlinelibrary.wiley.com/doi/10.1002/art.43255/abstract.

GLASSO networks were constructed for each timepoint and treatment outcome to explore and visualize the complex relationships between genes. Network analysis was performed to identify gene interactions and provide biologic contextualization, revealing crucial hub genes and novel biomarkers not highlighted by machine learning alone. This has the effect of enhancing predictive models through insights into gene‐gene interactions. A notable feature of the GLASSO method is its inference of sparse networks, leading to some genes not forming connections with others. In this context, “network” specifically refers to the connected component of the GLASSO network. Notably, for both timepoints, the networks in the nonresponder group included genes that were absent in the responders’ networks (Figures 4 and 5).

The baseline network exhibited a higher number of connections than the follow‐up network (Figure 4). Additionally, genes involved in the responders’ network at both timepoints were predominantly down‐regulated relative to those in the nonresponders’ network.

At baseline, eight genes unique to the nonresponders’ network were identified. Marginal zone B And B1 cell–specific protein 1 (*MZB1*) was a hub in both responder and nonresponder networks, exhibiting high‐degree centrality. A hub node is a vital point in a network with strong influence on the structure and function. The most informative gene in the baseline classifier was *CDC26P1*. This gene is currently annotated as a pseudogene, limiting interpretability. In the GLASSO networks, *CDC26P1* was a peripheral node in the baseline responder's network, connecting to only two other nodes. In contrast, it connected to five nodes in the nonresponders’ network, including two Ig genes and two long noncoding RNAs. *LMOD1*, found only in the nonresponders’ network, was connected to the hub node *MZB1*.

The follow‐up network was characterized by sparsity and contained fewer genes within the connected component for both treatment outcomes (Figure 5); 10 genes were uniquely present in the nonresponders’ network. Oleoyl‐acyl carrier protein hydrolase (*OLAH*) served as the pivotal hub node in the follow‐up network for both responders and nonresponders.

### *MZB1* association ADA

Given the importance of *MZB1* in the baseline RF model in predicting treatment outcome and its role as a hub node in the network as well as its biologic relevance to B cell and antibody production, we next tested if expression of this gene was predictive of production of ADA against adalimumab. The production of ADAs has been associated with poorer outcomes of adalimumab treatment.^29^ Of the 97 patient samples contributing data to the machine learning models, 80 patients had samples processed for ADA levels at one or more of the 3‐month, 6‐month, and 12‐month timepoints following treatment initiation. *MZB1* levels at baseline was found to be associated with the production of ADA in the Cox regression analysis (*P* = 5.3 × 10^−3^; hazard ratio, 1.58; 95% confidence interval, 1.14–2.17).

No significant immune‐cell associations identified from CIBERSORTx deconvolution analysis. No statistically significant associations were observed between CLR‐transformed cell‐type proportions and treatment response, with all *P* values exceeding 0.05 before multiple testing correction.

## DISCUSSION

The current study aimed to develop transcript‐based prediction models designed for repeated measures coupled with state‐of‐the‐art machine learning analyses to enable the identification of treatment response biomarkers before and during early treatment with adalimumab (Figure S11). Model overfitting was minimized by training models under robust nested cross‐validation and using a large RNA‐seq dataset (n = 100 patient samples at two timepoints) with transcriptome wide coverage. Using this approach, we identified relevant biomarkers of adalimumab response that now warrant further validation in independent samples.

The study underscored the associations of *HLA‐DQB1* and *HLA‐DQA1* with RA, with both genes up‐regulated in responders across both timepoints. It should be noted, however, that their importance in the RF models was relatively modest.

The identification of *MZB1* in the baseline responder model, a gene involved in B cell development and antibody production,^15^, ^32^ aligns with the functional enrichment results identifying adaptive immune response enriched for Ig genes. In the current study, pretreatment expression of *MZB1* was down‐regulated in samples from patients classified as responders following 6 months of treatment, which could indicate lower adaptive immune cell‐type composition, an association to responders as identified by Farutin et al.^33^ Given the development of ADAs observed in nonresponders to adalimumab,^29^ we explored the association of *MZB1* expression levels at baseline with ADA development and found that every unit increase of *MZB1* levels results in approximately 50% higher chance of developing ADAs. The lower expression of *MZB1* in responders may represent a reduced propensity for generating these neutralizing antibodies and may mechanistically explain the more favorable response to the treatment.

The differential expression analysis highlighted *LMOD1*'s potential as a biomarker of treatment response. This gene, involved in smooth muscle contraction,^34^ was up‐regulated in responders. In the network analysis, *LMOD1* was observed to interact with *MZB1*. Although there are no previous reports associating *LMOD1* with RA and immune response, there may be indirect associations that are not captured here. Finally, *FAM3B* was the most down‐regulated gene in nonresponders and ranked highly in the RF classifiers; however, its influence within the networks was limited.

*TRGV5*, although important in the follow‐up classifier, was absent in the follow‐up network, indicating its potential as a biomarker independent of gene interactions. *OLAH* was implicated in medium‐chain, fatty‐acid biosynthesis, which is the hub node of follow‐up network, and showed a notable upregulation in nonresponders with fold change of 1.15. *OLAH* is also known as *AURA1* and has been reported to be up‐regulated in bone marrow–derived mononuclear cells in patients with RA.^35^ This could be a proxy for active disease because lower expression was observed in responders compared with nonresponders after treatment. *OLAH* was connected to *ADAMTS2* in both responders and nonresponder networks; the ADAMTS family are involved in extracellular matrix remodeling and linked to arthritis.^36^ Similar to *OLAH*, *ADAMTS2* was up‐regulated in nonresponders with a fold change of 1.24. This suggests a potential link with active disease and *OLAH* and *ADAMTS2* levels because these two genes associated with disease activity at the same time and patients with higher expression levels were less likely to respond to treatment.

A strength of the current study was addressing the “*p* vs n” problem inherent in high‐dimensional transcriptomic datasets and including a feature selection step embedded within model development to avoid data leakage and prevent overfitting of the machine learning model. However, this approach limited the capture of gene interactions not identified through differential expression in the multivariate analysis, potentially affecting the performance of the resulting RF model. Furthermore, we chose the RF method for classification of treatment response because it is robust and performs well when analyzing tabular data.^37^ Both the baseline and follow‐up models achieved good performance across several classification metrics (Table S1). However, other methods, such as XGBoost,^38^ are available that may also give useful levels of performance. Future benchmarking studies would be needed to determine if other models can outperform the current approach.

This study leveraged transcriptomic profiles generated in whole‐blood samples, which are easily accessible and less invasive to capture compared with analysis of enriched cell types or sampling from joint tissues. However, this strategy also has limitations in that important signals may be averaged out in heterogeneous (whole‐blood) data; furthermore, the lack of data from synovial joint tissue means that important signals may have been missed in the current study. A further potential limitation to the current study was class imbalance, with a higher proportion of responders analyzed compared with nonresponders. This imbalance is reflective of the clinical reality in which a moderate‐to‐good response to adalimumab is seen in a majority of patients; however, this may have limited power to identify predictors of nonresponse. Because of the observational setting for this research, there is also a potential for bias in matching good responders and nonresponders that might have influenced the results. However, no major differences were observed between responder groups for the key variables included in the analysis. We chose to group good and moderate responders and compare with nonresponders, but an alternative approach would be to compare good responders and nonresponders as extreme phenotypes; however, that would reduce the sample size, potentially impacting power and would make the findings less generalizable in real‐world settings. Future research should focus on addressing these imbalances alongside validating findings in independent cohorts to strengthen the evidence base for the biomarkers identified. In addition, comparing results of RNA‐seq analysis of synovial tissue and fluid would also be useful. Further research to understand the link between *MZB1*‐related gene networks and ADA production is also now warranted. Indeed, elevated *MZB1* expression has previously been observed in peripheral B cells from patients with systemic lupus erythematosus and in synovial tissue from patients with RA with active disease compared with controls, suggesting a role for this protein in autoimmune diseases that are characterized by autoantibody production.^39^ The current study provides a comprehensive and nuanced first step in the understanding of the transcriptomic changes in peripheral whole blood associated with adalimumab treatment in RA, offering insights into potential biomarkers and gene interactions that could inform personalized treatment strategies, if replicated in independent cohorts.

## AUTHOR CONTRIBUTIONS

All authors contributed to at least one of the following manuscript preparation roles: conceptualization AND/OR methodology, software, investigation, formal analysis, data curation, visualization, and validation AND drafting or reviewing/editing the final draft. As corresponding author, Dr Plant confirms that all authors have provided the final approval of the version to be published and takes responsibility for the affirmations regarding article submission (eg, not under consideration by another journal), the integrity of the data presented, and the statements regarding compliance with institutional review board/Declaration of Helsinki requirements.

## Supporting information


**Disclosure form**.

**Appendix S1:** Supplementary Information
