# Supplementary material for: Identifying Predictive Biomarkers of Response in Patients With Rheumatoid Arthritis Treated With Adalimumab Using Machine Learning Analysis of Whole‐Blood Transcriptomics Data
Source: Arthritis Rheumatol. 2025 Aug 4;77(12):1663–72. doi: 10.1002/art.43255 (PMC12750119; doi:10.1002/art.43255)
Supplement: Supplementary file 2 — Appendix S1: Supplementary Information [file ART-77-1663-s002.docx]

Supplementary document for Machine Learning Analysis of Whole-Blood Transcriptomics Data in Rheumatoid Arthritis Patients Treated with Adalimumab Identifies Predictive Biomarkers of Response

# Supplementary Methods

## Gene Set Enrichment Analysis (GSEA)

To investigate the molecular pathways distinguishing treatment responders from non-responders, we performed Gene Set Enrichment Analysis (GSEA) on ranked gene expression profiles. A total of 49 curated gene sets were evaluated for enrichment in responders and non-responders, as well as pre-treatment and post-treatment. Enrichment was assessed based on the Normalized Enrichment Score (NES), with significance determined at a false discovery rate (FDR) threshold of 0.25 and nominal p-value thresholds of 0.01 and 0.05. As this was done with results from paired samples, GSEA pre-ranked approach was use. The ranking was carried out using z-scores of the coefficients for response and timepoint for responders vs non-responders and pre-treatment vs post-treatment respectively.

# Supplementary Results


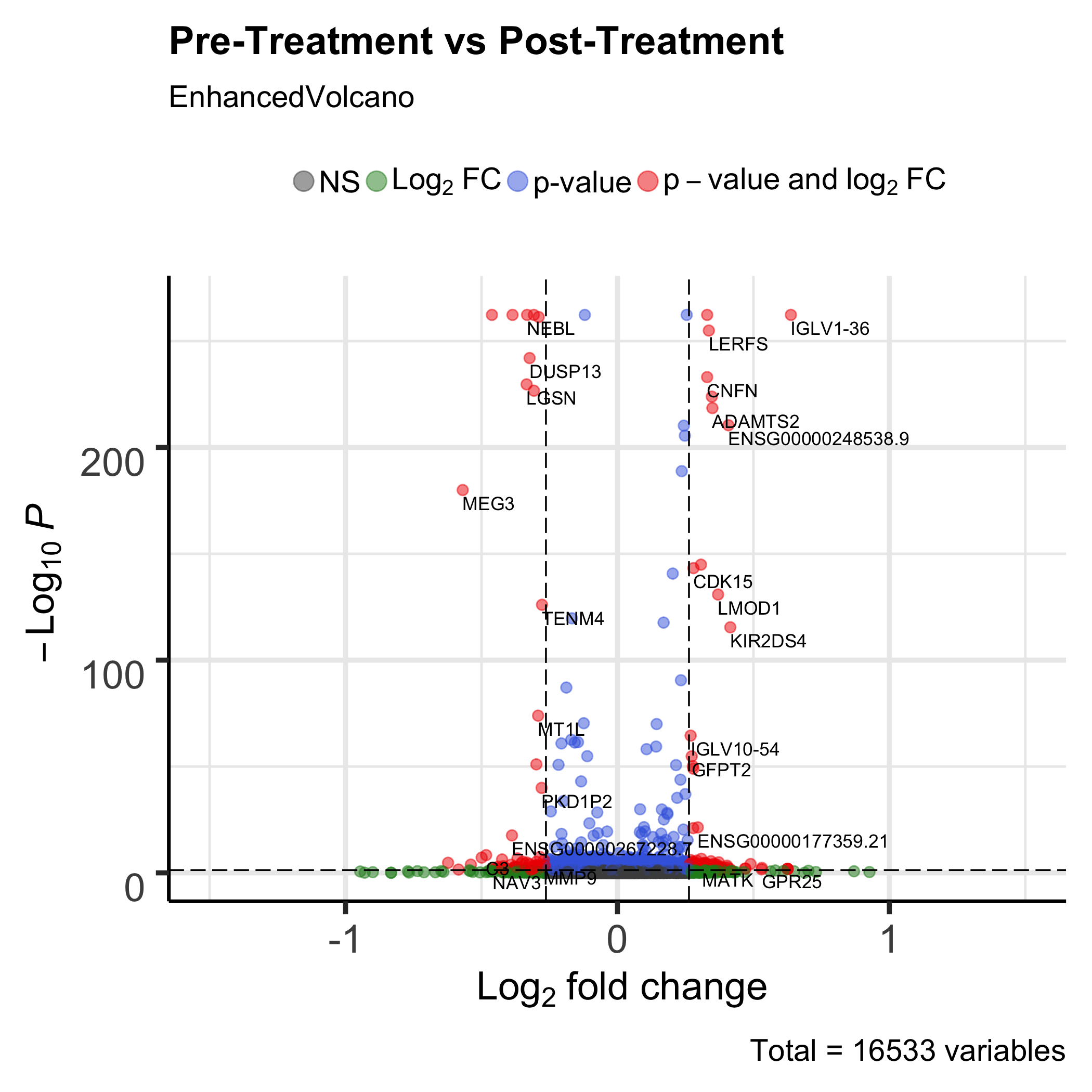


Figure S1: Volcano plot of pre-treatment vs post-treatment differential expression analysis.


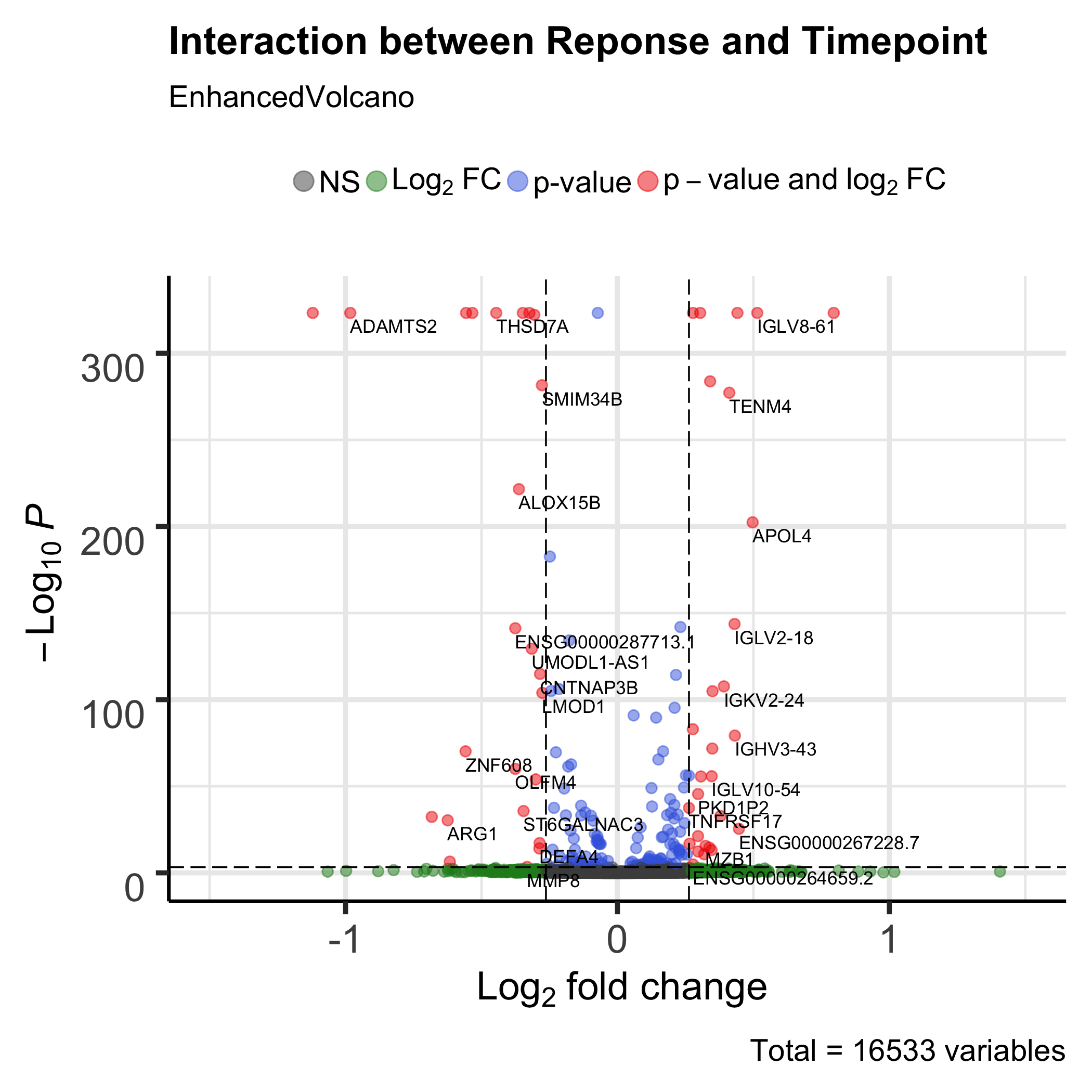


Figure S2: Volcano plot of the interaction analysis between time of treatment and response outcome.

## GSEA results

### Responders vs non-responders

In responders, 30 out of 49 gene sets were upregulated. Among these, two gene sets reached statistical significance at FDR<0.25, which is the recommend threshold for GSEA. These gene sets are “Genes down-regulated in response to ultraviolet (UV) radiation” (Fig S3) and “Genes involved in protein secretion pathway” (Fig S4). These results suggest that specific these biological pathways are more active in responders compared to non-responders.

In non-responders, 19 out of 49 gene sets were upregulated. However, none of these gene sets reached significance at FDR<0.25, indicating a weaker pathway enrichment profile in non-responders compared to responders.


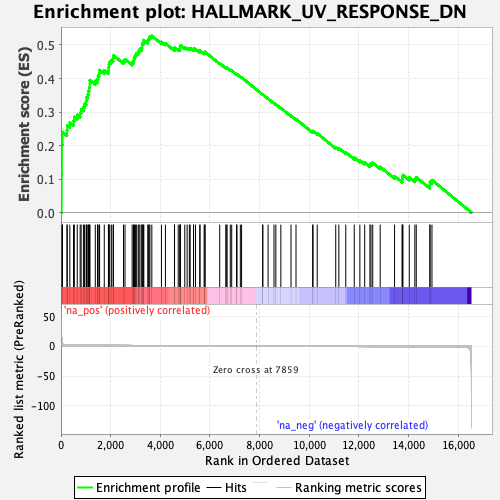


Figure S3: Enrichment plot for “Genes down-regulated in response to ultraviolet (UV) radiation” in responders.


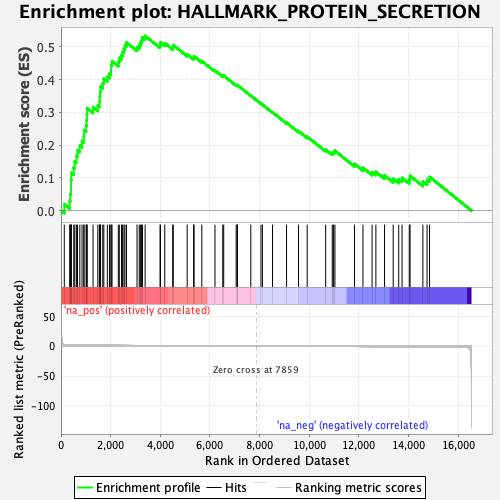
Figure S4: Enrichment plot for “Genes involved in protein secretion pathway” in responders.

### Pre-treatment vs Post Treatment

In pre-treatment samples, 19 out of 49 gene sets were upregulated. No gene sets reached statistical significance at FDR<0.25, suggesting limited pathway activation before treatment.

In post-treatment samples, 30 out of 49 gene sets were upregulated. Four unique gene sets were significantly enriched at FDR<0.25. These results indicate significant activation of biological pathways following treatment. The enriched gene sets are “A subgroup of genes regulated by MYC” (Fig S5), “Genes encoding cell cycle related targets of E2F transcription factors” (Fig S6), “Genes down-regulated in response to ultraviolet (UV) radiation”, (Fig S7) and Genes encoding proteins involved in oxidative phosphorylation (Fig S8).


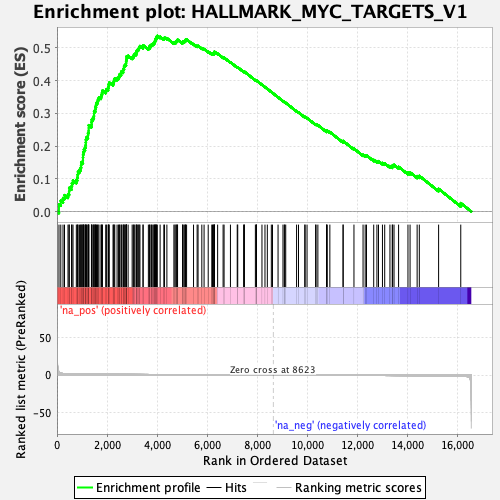


Figure S5: Enrichment plot for “A subgroup of genes regulated by MYC” in post-treatment.


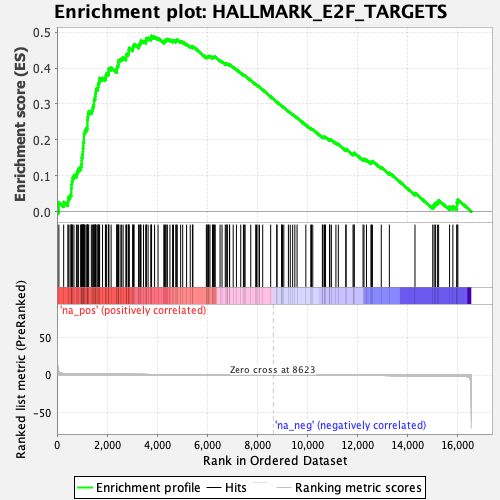


Figure S6: Enrichment plot for “Genes encoding cell cycle related targets of E2F transcription factors” in post-treatment.


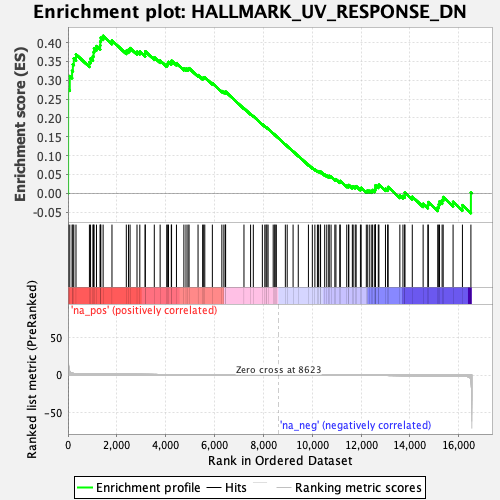


Figure S7: Enrichment plot for “Genes down-regulated in response to ultraviolet (UV) radiation” in post-treatment.


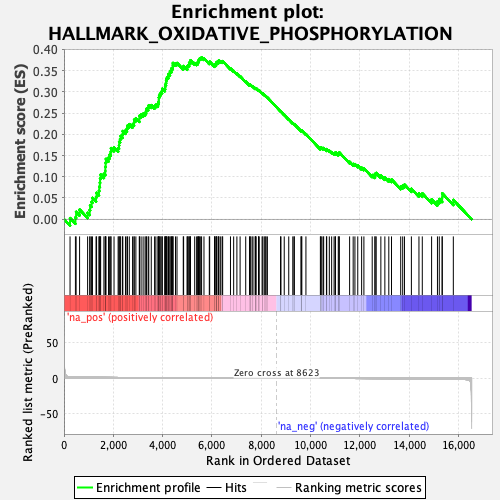


Figure S8: Enrichment plot for “Genes encoding proteins involved in oxidative phosphorylation” in post-treatment.


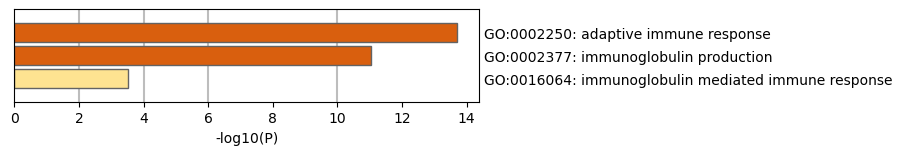


Figure S9: Functional enrichment outcome from the top 30 genes of baseline classifier using Metascape.


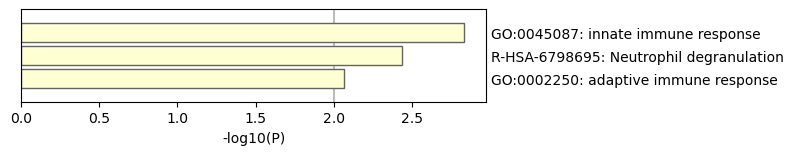


Figure S10: Functional enrichment outcome from the top 30 genes of follow-up classifier using Metascape.
